# Complete supine PCNL: ultrasound vs. fluoroscopic guided: a randomized clinical trial

**DOI:** 10.1590/S1677-5538.IBJU.2014.0291

**Published:** 2016

**Authors:** Siavash Falahatkar, Aliakbar Allahkhah, Majid Kazemzadeh, Ahmad Enshaei, Maryam Shakiba, Fahimeh Moghaddas

**Affiliations:** 1 Urology Research Center, Guilan University of Medical Sciences, Guilan, Iran

**Keywords:** Nephrostomy, Percutaneous, Ultrasonography, Fluoroscopy, Supine Position

## Abstract

**Introduction and Hypothesis:**

To compare complications and outcomes of complete supine percutaneous nephrolithotomy (csPCNL) with ultrasound guided and fluoroscopically guided procedure.

**Materials and Methods:**

In this randomized clinical trial study from January 2009 to September 2010, 26 of 51 patients with renal stones underwent csPCNL with ultrasonographic guidance in all steps of the procedure (group A), and the other 25 patients underwent standard fluoroscopically guided csPCNL (group B). All of the patients underwent PCNL in the complete supine position. Statistical analysis was performed with SPSS16 software.

**Results:**

Mean BMI was 28.14 in group A and 26.31 in group B (p=0.30). The mean stone burden was 26.48 and 30.44 in groups A and B, respectively (p=0.20). The stone free rate was 88.5% in group A and 75.5% in group B, that was no significant (p=0.16). Overall 2 patients (7.7%) in group A and 6 patients (24%) in group B had complications (p=0.11). Mean operative time in group A was 88.46 minutes, and in group B it was 79.58 minutes (p=0.39). Mean hospital stay was 69.70 and 61.79 hours in group A and B, respectively (p=0.22). There was no visceral injury in groups.

**Conclusions:**

This randomized study showed that totally ultrasonic had the same outcomes of fluoroscopically csPCNL. Ultrasonography can be an alternative rather than fluoroscopy in PCNL. We believe that more randomized studies are needed to allow endourologists to use sonography rather than fluoroscopy in order to avoid exposition to radiation.

## INTRODUCTION

Percutaneous nephrolithotomy (PCNL) is a common method for treatment of kidney stones (1, 2). All of the steps in PCNL should be performed with proper image guidance. The imageless PCNL should never be applied because it is dangerous to vital structures (3).

The popular imaging of PCNL is fluoroscopy, so the patient and surgical team are exposed to some level of radiation by fluoroscopy during PCNL. The side effects of extensive radiation are well known. Thus, the ultrasound-guided PCNL can be an alternative method to decrease the radiation exposure hazard to the surgeon (4-6).

The purpose of present study is to compare complications and outcomes in patients who underwent complete supine percutaneous nephrolithotomy (csPCNL) with these two methods and to share the experience of the authors with totally ultrasound-guided csPCNL procedure with the urological community.

## MATERIAL AND METHODS

In this randomized clinical trial study from January 2009 to September 2010, 51 patients with renal stones were selected for csPCNL. All participants were informed about the surgical method and consent. We used totally ultrasonographic guidance in all steps of the procedure during csPCNL in 26 of our patients (group A), whereas the other 25 patients underwent standard fluoroscopically guided csPCNL (group B). All patients in both groups performed PCNL in the complete supine position without any towel under the patient's flank and with no change in leg position. For all patients, routine blood and urine tests, coagulation profile and imaging series, including intravenous urogram and ultrasonography, were carried out and medical conditions were studied.

Inclusion criteria were patients with single large pelvic stone, lower caliceal stone, stones in the pelvis and lower calyx, middle caliceal stones, and non-opaque stones (staghorn stones) with hydronephrosis.

Exclusion criteria's in this study were multiple stones in multiple calyxes, staghorn stones (except non-opaque stones), urinary tract anomalies, single kidney and morbid obesity and non-opaque stones (staghorn stones) without hydronephrosis.

All of the patients underwent general anesthesia, and a 5F ureteral catheter was placed transurethrally for injection of saline or contrast media. Injection of saline obtained mild dilatation of collecting system and this was useful especially for the totally ultrasound-guided PCNL group.

In group A, ultrasonography was used to observe the location of the kidney, needle entrance point, urinary tract dilatation and to check for residual stone at the end of csPCNL. Because the Rouch guidewire is more rigid, and in order to not miss the access, we used this type of guidewire, although the guidewire was clearly visible but the Amplatz dilatators and the Amplatz sheath were not exactly visible by ultrasonography.

In group B, we performed all the above steps of csPCNL with the guidance of fluoroscopy. Our technique was a one-shot dilatation in both groups.

In this study, the items including side of renal unit, stone burden, stone-free rate, complications (extravasation, colon injury, fever, etc.), and the history of previous open renal surgery or previous ESWL, mean hospital stay, mean operative time, body mass index (BMI), serum creatinine before the operation, and hemoglobin before and after the csPCNL were studied.

In group A, after removal of the stone(s), ultrasonography was used to detect any residual stones, hematoma, or extravasation of urine outside of the kidney.

In the fluoroscopic group, residual stones and extravasation were checked by fluoroscopy. We performed tubeless PCNL except in patients with severe extravasation, ureteral obstruction, severe hemorrhage, or large residual stone.

Statistical analysis was performed with SPSS16 software. A P value of less than 0.05 was considered statistically significant.

This study was approved by ethical review committee of Guilan University of Medical Science and the trial registered at http://www.irct.ir (IRCT138805251853N3).

## RESULTS

Total number of patients in both groups was 51 (26 patients in group A and 25 patients in group B). Demographic data and stone characteristics of two groups are shown in Table-1. In group A, mean age was 48.41 years and in group B it was 51.17 years (p=0.46). Mean BMI was 28.14 in group A and 26.31 in group B (p=0.30). The mean hemoglobin level before operation was 12.81 and 13.38 in groups A and B, respectively (p=0.23). The mean stone burden was 26.48 and 30.44 in groups A and B, respectively (p=0.20). The stone burden was detected on the basis of maximum diameter of stones on the KUB or ultrasonography. 4 patients (15.4%) in group A and 7 patients (28%) in group B had coexisting disease (p=0.44). All of the patients underwent general anesthesia and the access was sub costal in all patients. Intra and postoperative parameters of the two groups are shown in Table-2. The stone free rate was 88.5% in group A and 75.5% in group B, that was no significant (p=0.16). Overall, 2 patients (7.7%) in group A and 6 patients (24%) in group B had complications (p=0.11). In group A, 1 patient (3.8%) had fever, and in group B, 4 patients (16%) needed transfusion and 2 patients (8%) had fever (Grade I and II of the Clavien Classification of Surgical Complications). Mean operation time in group A was 88.46 minutes, and in group B, it was 79.58 minutes (p=0.39). Mean hospital stay was 69.70 and 61.79 hours in groups A and B, respectively (p=0.22). There was no complications compatible with Grade III to V of the Clavien Classification of Surgical Complications in both groups.

**Table 1:** This table showed the demographic data of two groups according to method of study.

Total N		Ultrasonographic Group	Fluoroscopic Group	P_Value
		26	25	-
**Sex**				
	Male (%)	17(65.4)	15(60)	0.69
	Female (%)	9 (34.6)	10(40)	
Age (Year)		48.41	51.17	
	Mean (SD)	(13.22)	(11.82)	0.46
BMI (Kg/m^2^)		28.17	26.31	
	Mean (SD)	(4.17)	(5.88)	0.30
Serum Cr. before the Operation,		1.45	1.16	
	Mean (SD)	(1.60)	(0.28)	0.38
Hb before the Operation,		12.81	13.38	
	Mean (SD)	(1.78)	(1.56)	0.23
Stone Size (mm),		26.48	30.44	
	Mean (SD)	(10.90)	(11)	0.20
Number of Stones,		1.42	1.58	
	Mean (SD)	(0.50)	(0.50)	0.26
**Side,n(%)**				
	Right	15(57.7)	17(68)	0.51
	Left	10(38.5)	8(32)	
**Co-existing Disease, n (%)**				
	Yes	4(15.4)	7(28)	0.44
	No	22 (84.6)	19(76)	
**Previous open or percutaneous surgery, n (%)**				
	Yes	6 (23.1)	7(28)	0.68
	No	20 (76.9)	18(72)	
**Previous ESWL, n (%)**				
	Yes	11 (42.3)	13(52)	0.48
	No	15(57.7)	12(48)	

**Table 2:** This table showed the comparison of results after the procedure between two groups.

Total N		Ultrasonographic Group	Fluoroscopic Group	P_Value
		26	25	-
**Stone free rate (%)**				
	Stone free	20 (77)	17 (71)	0.16
	Residual stone<5mm	3 (11.5)	1 (4.5)	
	Residual stone<5mm	3 (11.5)	6 (27.3)	
**Complications**				
	Yes	2 (7.7)	6 (24)	0.1
	No	24 (92.3)	19 (76)	
**Nephrostomy tube**				
	Yes	2 (8.7)	1 (4.3)	0.55
	No	21 (91.3)	22 (95.7)	
Duration of access to target calyx (sec)		14.36	14.78	0.08
	Mean (SD)	(14.84)	(25.54)	
Duration of entrance to target calyx (sec)		84.87	41.22	0.07
	Mean (SD)	(112.83)	(48.51)	
Duration of 9Fr dilator dilatation (sec)		22.48	23.39	0.78
	Mean (SD)	(26.7)	(37.7)	
Duration of Amplatz dilator dilatation (sec)		32.72	15.57	0.77
	Mean (SD)	(82.45)	(15.94)	
Duration of Amplatz sheath insertion (sec)		17.46	12.41	0.28
	Mean (SD)	(26.72)	(15.67)	
Hb Drop after the operation		1.11	1.14	
	Mean (SD)	(1.35)	(1.52)	0.93
Operating time, min		88.46	79.58	
	Mean (SD)	(39.49)	(32.6)	0.39
Hospital stay, hour		69.70	61.79	
	Mean (SD)	(18.87)	(25.22)	0.22
Extravasation (%)		0	0	-
Pseudo aneurism (%)		0	0	-
Fever, n (%)		1(3.8)	2(8)	-
Colon injury (%)		0	0	-

## DISCUSSION

The scope of endourology despite its short age has been widened. The first step in percutaneous procedures is to access to the collecting system, usually performed by fluoroscopy, ultrasonography, or computed tomography (CT) guidance (7-9).

To reduce the risk of radiation exposure, using ultrasonography for PCNL can be an alternative imaging method to fluoroscopy as the first and standard imaging technique (10, 11).

Some studies reported that PCNL under ultrasonography guidance in the flank or prone position has high success rates and limited complications and can be a safe and effective alternative to fluoroscopy in experienced hands (11-18).

Ultrasound-guided PCNL without fluoroscopy has some advantages and disadvantages. Advantages: Avoidance of X-ray exposure, no necessity of lead shield, all organs on the way of access are visible, search for residual stones at the end of the procedure especially for non-opaque stones. Disadvantages: Endourologists are unfamiliar with ultrasonography, and poor echogenicity of the Amplatz dilatator and Amplatz sheath (11, 12, 19, 20).

Nowadays, PCNL is considered a generally safe management option with a low incidence of complications and is the method of choice for treatment of renal stones (11, 21, 22).

PCNL is done in the prone, flank, semi-supine, and csPCNL positions. We performed csPCNL in our patients due to better control of the airway, better tolerance for patients especially with cardiopulmonary disease, easier to perform ureteroscopy or TUL, better drainage and evacuation of stones by the Amplatz sheath, possibility to change regional anesthesia to general anesthesia, and probably less risk of colon injury. These are some advantages of csPCNL (13).

Because of levitation of the colon in the abdominal cavity in the supine position it is less affected to injury when puncture site is in the posterior axillary line (23).

The access to the kidney is important in PCNL and usually is performed by fluoroscopy, ultrasonography, or computed tomography guidance with rates of success of 86.7-100% (9, 11, 20, 24-26). The success rate in achieving access in our study was 100% in both groups. The gaining access to the collecting system under ultrasonographic guidance was similar to the fluoroscopic-guided access.

Some studies showed that the stone-free rate in percutaneous nephrolithotomy with ultrasonography guidance varied from 66.6 to 94.7% (5, 7, 12, 18, 20, 27). Other studies showed that primary stone-free rate and total stone-free rate with ultrasound-guided percutaneous nephrolithotomy were 45.7 – 69.6% and 82.6 – 96.5%, respectively (21, 26). In our study, similar to the others, the stone-free rate was 88.46% and 72%, without any significant statistical difference in groups A and B, respectively (p=0.16).

The mean operative time was 120±68 minutes (range 45-350) in one study. The real-time ultrasound can be used to guide the percutaneous puncture (26). In another study mean (range) of operative time was 111 (70-180) minutes. They emphasized that ultrasonographic - guided PCNL is feasible but fluoroscopy must be present in the operating room (27). Mean operative time was reported as 88.92 and 79.28 minutes in sonographic and fluoroscopic groups, respectively (20). In the current study mean operative time was similar to other studies without any significant statistical difference (p-value=0.39).

Hospital stay was 3.6 days (range 2-8 days) in one study and other studies reported 2.7 to 4.1 days (5, 12, 20 24, 27). In our study, hospital stay was similar to other studies without any significant difference (p=0.22).

Although we had seen more complications in fluoroscopic group, they were not significant.

In this study we found no extravasation in both groups. This result was similar to others (20, 21). Some studies reported 4-9% with postoperative fever (12, 18). Other studies reported 26.3-27.6% postoperative fever and the patients responded to antibiotics (21, 27). In this study fever had no effect on the results of our study. All of the patients with fever were cured with appropriate antipyretics and antibiotics. Septic shock was not a major complication in our patients.

In other studies, like ours, no severe complications such as colon damage, pneumothorax or hydrothorax or any adjacent injuries occurred (24, 26). We had no patient with injury to adjacent organs during csPCNL till now.

Totally ultrasound-guided PCNL is feasible and safe in patients with a history of renal surgery (28).

In current study mean BMI was similar to the other studies without any significant statistical difference between the two groups (p-value=0.3); therefore, BMI had no effect on the results of our study. We achieved access in all patients and we believe that ultrasound-guided csPCNL in obese patients is more difficult but it is safe and feasible. Sometimes it was imperative to draw up the fatty abdomen with a strip band for preventing any encumbrance during the procedure.

PCNL is feasible and safe in the supine position (13, 29-32).

## CONCLUSIONS

This randomized study showed that totally ultrasonic csPCNL had the same outcomes of fluoroscopically-guided csPCNL. We believe that more randomized studies are needed to allow endourologists to use sonography rather than fluoroscopy to avoid exposing the radiation.
